# P05-06 Composition of physical behaviors at work and risk of sick leave due to musculoskeletal pain

**DOI:** 10.1093/eurpub/ckac095.073

**Published:** 2022-08-29

**Authors:** David Hallman, Nidhi Gupta, Andreas Holtermann

**Affiliations:** 1 Occupational health sciences and psychology, University of Gävle, Gavle, Sweden; 2 National Research Centre for the Working Environment, Copenhagen, Denmark, National Research Centre for the Working Environment, Copenhagen, Denmark, Copenhagen, Denmark

**Keywords:** Accelerometer, Compositional data analysis, Occupational health, Pain

## Abstract

**Background:**

Sick leave due to musculoskeletal pain is common in the workforce. Time use in physical behaviors at work such as sitting, standing, low- (LIPA) and moderate-to-vigorous physical activity (MVPA) may impact on sick leave due to pain. However, studies addressing this relationship using technical measures of physical behaviors are scarce. The aim was to investigate the association between time-use compositions of physical behavior at work and sick leave trajectories due to musculoskeletal pain over one year.

**Methods:**

We analyzed data of 981 workers in a Danish cohort (DPHACTO 2012-2014). We assessed physical behaviors at work at baseline using thigh-worn accelerometers, and classified behaviors at work as sitting, standing, LIPA, and MVPA. Over 1 year follow-up, workers reported sick leave days due to musculoskeletal pain using text messages at 4-week intervals (14 waves). We used Latent class growth analysis to distinguish sub-groups with different trajectories of sick leave. We analyzed associations between time-use in physical behaviors and sick leave trajectories using multinomial regression analysis with adjustment for age, gender, BMI, smoking, and accelerometry-measured physical activity during leisure. Compositional data analysis was used to account for the co-dependency of different behaviors.

**Results:**

We identified four distinct trajectories of sick leave due to pain over one year as follows: no sick days (prevalence 76%), few days-increasing trajectory (19%), some days-decreasing trajectory (3%), and some days-increasing trajectory (2%). Spending more time in sitting relative to the other behaviors was negatively associated with few days-increasing trajectory of sick leave (p > 0.001), while time in LIPA was positively associated with some days-increasing trajectory of sick leave (p = 0.001). Reallocating 60 min/day from sitting to other behaviors at work predicted a 22% increased likelihood of few days-increasing trajectory of sick-leave. In contrast, reallocating 30 min/day from LIPA to other behaviors at work predicted a 57% decreased likelihood for some days-increasing trajectory.

**Conclusion:**

We found that compositions with more sitting relative to the other behaviors had lower odds for the trajectory with increasing sick leave due to pain, while compositions with more LIPA had higher odds. This may have implications for prevention of pain-related sick leave in workers.

